# Supplementary material for: Migratory convergence facilitates cultural transmission of humpback whale song
Source: R Soc Open Sci. 2019 Sep 4;6(9):190337. doi: 10.1098/rsos.190337 (PMC6774987; doi:10.1098/rsos.190337)
Supplement: Supplementary Information [file rsos190337supp1.pdf]

ROYAL SOCIETY
OPEN SCIENCE

R. Soc. open sci.
doi:10.1098/not yet assigned

Supporting Material: Migratory convergence facilitates cultural transmission of humpback whale song

Clare Owen, Luke Rendell, Rochelle Constantine, Michael J. Noad, Jenny Allen, Olive Andrews, Claire Garrigue, M. Michael Poole, David Donnelly, Nan Hauser, Ellen C. Garland.

1. Supplementary Results

1.1. Description of song types
Song type 1 contained eight themes and 15 phrase types (figure 1, table S2). Six phrase types were present in New Caledonia (7A, 9A, 11A, 12A-B, 14A), five in Tonga (7C, 8A-B, 9A, 10A), 13 in the Cook Islands (7A-C, 8A-B, 9A, 10A & C, 11A, 12A-B, 13A, 14A) and ten in French Polynesia (7A-7C, 8A-B, 9A, 10A-C, 11A). Song type 1 was the dominant song in the central Pacific (*i.e.,* the Cook Islands and French Polynesia) and was highly complex. It contained two versions and a high level of individual and population variation.

Song type 2 contained six themes and 18 phrase types (figure 1, table S2). Of these 18 phrase types, 17 were present in New Caledonia (1A-B, 2A-B, 3A-D, 4A-C, 5A-C, 6A-C), 15 in Tonga (1A-B, 2A-B, 3A & C, 4B-C, 5A-B, 6A-C), 13 in Niue (1A-B, 2A-C, 3A, 4A-B, 5A-5B, 6A-C), and ten in the Cook Islands (1A-B, 2B-C, 3C, 5B-C, 6A-C). Song type 2 was most prevalent in the west (New Caledonia, Tonga and Niue). The song was acoustically complex and contained a large number of themes and phrases. Although only one version of this song was present, there was evidence of very fine-scale individual and population level variation.

Song type 3 contained three themes and five phrase types, all of which were present only in eastern Australia (15A-B, 16A-B, 17A). Song type 3 was very simple as it contained five phrases which were highly stereotyped with little variation both within and among the different singers.

The Kermadec recordings contained 22 (of the 38) phrase types including all of the phrases present in song 2 (1A-B, 2A- 2C, 3A-D, 4A-C, 5A-C, 6A-C) and three from song type 1b (7A, 12B, 14A). A single phrase type (8C) was only recorded in the Kermadecs and was assigned to song type 1 (based on quantitative LSI clustering).

1.2. Fine-scale theme similarity of Kermadec singers with the wintering grounds
Hierarchical clustering of theme 1 divided the data into 13 stable clusters (AU>95%; figure 3*a*). Three clusters paired a singer from the Kermadecs with singers from Tonga, resulting in seven Kermadec singers being assigned to Tonga, and one cluster paired a Kermadec singer with Niue (AU *p*-value >95%; figure 3*a*, table 1). Phrases from theme 3 were divided into 17 stable clusters (AU *p*-value >95%; figure 3*b*). Here, five singers from the Kermadecs were paired with Tonga and three with New Caledonia (AU *p*-value >95%; figure 3*b*, table 1); Kermadec singers were not paired with a specific wintering ground on the remaining clusters.

Author for correspondence: Ellen Garland (ecg5@st-andrews.ac.uk).
Present address: School of Biology, University of St. Andrews, St. Andrews, KY16 8LB, United Kingdom

2. Supplementary Tables

Table S1. Song recordings included in each level of the analysis.

Location	Total # singers	Total time *(hh:mm:ss)*	Recording date range (dd/mm/yy)	Unit-level transcription		Theme-level transcription		Song types present
				# Singers	*# Complete phrases*	*# Singers*	*# Song sequences*	
Kermadec Islands	39	03:57:36	29/09/15 - 08/10/15	39	631	17	31	1, 2
Eastern Australia	11	02:10:00	01/10/15 - 15/10/15	11	303	6	15	3
New Caledonia	11	05:51:25	18/07/15 - 09/09/15	11	1182	9	43	1, 2
Tonga	8	01:20:06	21/08/15 - 14/09/15	8	229	7	11	1, 2
Niue	7	01:24:01	22/08/15 - 01/09/15	7	143	6	9	2
Cook Islands	8	01:42:14	21/08/15 - 20/09/15	8	444	6	16	1, 2
French Polynesia	7	02:57:49	24/09/15 - 04/10/15	7	570	6	25	1
**Total**	**91**	**19:23:11**	**Jul-Oct 2015**	**91**	**3502**	**57**	**150**	

Table S2. Additional singer information corresponding to table 1 Kermadec singers including recording number, date and duration, singer number and number of phrases included in analysis from each 'singer'. When multiple singers were present in a recording or a singer was silent for more than three minutes before the song continued, it was not always possible to confirm the same singer was resuming. To avoid ambiguity, the subsequent phrases were labelled with a letter (*e.g.,* KI01S1a) and the strings of phrases were analysed separately. Only singers that sung >1 phrase type are included in the table.

Recording #	Recording date	Recording duration (mm:ss)	Kermadec singer #	# phrases
KI15-01	29-Sep-2015	16:36	KI01S1	5
			KI01S1a	8
			KI01S1b	5
KI15-02	29-Sep-2015	09:14	KI02S1a	5
			KI02S2	2
			KI02S3	5
KI15-03	29-Sep-2015	11:01	KI03S3	5
			KI03S4	7
			KI03S5	2
KI15-04	30-Sep-2015	13:31	KI04S1	3
			KI04S1b	4
			KI04S2	4
			KI04S3	5
KI15-05	01-Oct-2015	32:00	KI05S1	6
			KI05S2	11
			KI05S3	5
KI15-06	02-Oct-2015	28:01	KI06S1	10
KI15-10	04-Oct-2015	09:16	KI10S1	5
			KI10S2b	3
KI15-11	04-Oct-2015	06:48	KI11S1	2
KI15-12	08-Oct-2015	39:26	KI12S1	3
			KI12S2	5
			KI12S3	7
			KI12S4	10
			KI12S5	10
			KI12S6	6
			KI12S7	5
KI15-13	08-Oct-2015	33:28	KI13S1	11

	KI15-14	08-Oct-2015	30:17	KI14S1	10
				KI14S1a	7
				KI14S2	3
**Total**	**11**		**3:49:38**	**31 ‘singers’**	**179**

Table S3. Most representative (set median) sequence of units for every phrase type for all song types included in the study (weighted analysis). Song type 1 included themes 7-14, song type 2 themes 1-6, and song type 3 themes 15-17. KI=Kermadec Islands, EA=eastern Australia, NC=New Caledonia, TO=Tonga, NI=Niue, CI=Cook Islands, FP=French Polynesia. Each letter or combination of letters represents a unit type (see table S4 for unit names). A comma separates units.

Song type	Theme	Phrase type	Population(s) present	Set median unit sequence
1	7	A	KI, NC, CI, FP	DGR(L), UC(S)-C(H), DC(S), UC(S)-C(H)
		B	TO, CI, FP	BE, TI(A), AS(S)-AW, AS(S), AS(S)-AW
		C	TO, CI, FP	BE-TI(A), UC(S)-C(H), UC(S)-C(H)(S), C(H)
	8	A	TO, CI, FP	AGR(H), AGR(H), SQ, BP, ASQ, BP, ASQ, BP, ASQ, BP, AGR(H), AGR(H), SQ, BP, ASQ, BP, ASQ, BP, ASQ, BP, AGR(H), BP(A)
		B	TO, CI, FP	AGR(H), AGR(H), AC(H), SQ, BP(A), AGR(H), AGR(H), AC(H), SQ, BP
		C	KI	AGR(H), AGR(H), BP, BP(A), AGR(H), AGR(H), BP, BP(A)
	9	A	NC, TO, CI, FP	GW(L), EE(L)
	10	A	TO, CI, FP	AGR, P, AGR, QK, BP(A), BP(A), AGR, BP(A)
		B	FP	AGR, P, AGR, QK, BP, AGR, BP(A)
		C	CI, FP	AGR, P-AGR, BP(A), BP(A), P-AGR, BP(A)
	11	A	NC, CI, FP	SC(L), LWP, P-LWP, P-LWP, DGR(S), P-LWP, DGR(S)
	12	A	NC, CI	DGR, GR(S)-SQ-MODC, GR(S)-SQ, GR(S)-SQ-MODC, GR(S)-SQ, GR(S)-SQ-MODC
		B	KI, NC, CI	DGR, MODGR, GR(S)-NSQ, MODGR
	13	A	CI	DGR, P-AGR, WP, P-AGR, WP, P-AGR, WP, P-AGR, WP, P-AGR, WP
	14	A	KI, NC, CI	DGW, WP, AW, DW, WP, AW, MODW
2	1	A	KI, NC, TO, NI, CI	M, TI(A), TI(A), TI(A), TI(A), TI(A)
		B	KI, NC, TO, NI, CI	M, P(A), P(A), P(A), P(A)
	2	A	KI, NC, TO, NI	M, AGR(S)-DW, BA, BA, BA, AGR(S)-DW, BA, BA, BA, AGR(S)-DW, BA(H), BA(H), BA(H), AGR(S)-MODW
		B	KI, NC, TO, NI, CI	M, AGR(S)-DW, BA(P), BA(P), AGR(S)-DW, BA(P), BA(P), AGR(S)-MODW
		C	KI, TO, NI, CI	M, AGR(S)-DW, BA(P), AGR(S)-MODW, BA(P), AGR(S)-MODW
	3	A	KI, NC, TO, NI	M, GW, GW, GW, GW, GW, GW
		B	KI, NC	M, SC, SC, SC, SC, SC
		C	KI, NC, TO, CI	M, GW(MOD)(L), GW(MOD)(L)
		D	KI, NC, NI	M, SC(L), SC(L)
	4	A	KI, NC, TO, NI	M, E, E, E, E, E, E, E, E, E, E, E, E, E, E, E, E, E, E, E, E, E, E, E, E, E, E, E, E, E, E, E, E, E, E, E
		B	KI, NC, TO, NI	M, SC-E
		C	KI, NC, TO, NI	M, GR(S), E, E, E, E, E, E, E, E, E, E, E, E, E, E, E, E, E, E, E, E, E, E, E, E, E, E, E, E, E, E, E, E, E, E, E
	5	A	KI, NC, TO, NI	M, BE, BE
		B	KI, NC, TO, NI, CI	M, P(MOD)(L), P(MOD)(L)
		C	KI, NC, CI	M, TI(MOD)(L), TI(MOD)(L)
	6	A	KI, NC, TO, NI, CI	M, AM, AM, AM, AM
		B	KI, NC, TO, NI, CI	M, PE-AM, PE-AM, PE-AM, PE-AM
		C	KI, NC, TO, NI, CI	M, AM(P), AM(P), AM(P)(S), AM(P), AM(P), AM(P)
3	15	A	EA	AGR, AGR(L), MODC(H)(L), AC(H)
		B	EA	AGR, BE, MODC(H)(L), AC(H)
	16	A	EA	MODGR, DGR, GT, BP, GT, BP, GT, BP, GT, BP, GT, BP, DGR, GT, BP, GT, BP, GT, BP, GT, BP, GT, BP
		B	EA	MODGR, DGR, NGR(S), NGR(S), NGR(S), NGR(S), DGR, NGR(S), NGR(S), NGR(S), NGR(S)
	17	A	EA	AM(L), GR-AW, GR-WHOP, GR-WHOP, GR-AW, GR-WHOP, GR-WHOP, GR-AW

Table S4. Unit names for all unit abbreviations in table S2.

Unit code	Unit name
AC(H)	High ascending cry
AGR	Ascending groan
AGR(H)	High ascending groan
AGR(L)	Long ascending groan
AGR(S)-DW	Short ascending groan – descending whistle
AGR(S)-MODW	Short ascending groan – modulated whistle
AM	Ascending moan
AM(L)	Long ascending moan
AM(P)	Pulsed ascending moan
AS(S)	Ascending shriek
AS(S)-AW	Ascending shriek – ascending whistle
ASQ	Ascending squeak
AW	Ascending whistle
BA	Bark
BA(H)	High bark
BA(P)	Pulsed bark
BE	Bellow
BE-TI(A)	Bellow – ascending trill
BP	Flat beep
BP(A)	Ascending beep
C(H)	High, flat cry
DC(S)	Short descending cry
DGR	Descending groan
DGR(L)	Long descending groan
DGR(S)	Short descending groan
DGW	Descending growl
DW	Descending whistle
E	E sound
EE(L)	Long double e sound
GR(S)	Short, flat groan
GR-AW	Flat, groan – ascending whistle
GR-WHOP	Flat groan - whop
GR(S)-NSQ	Short, flat groan – N-shaped squeak
GR(S)-SQ	Short, flat groan - flat squeak
GR(S)-SQ-MODC	Short, flat groan – flat squeak – modulated cry
GT	Grunt
GW	Flat growl
GW(L)	Long growl
GW(MOD)(L)	Long modulated growl
LWP	Low whoop
M	Flat moan
MODC(H)(L)	High, long modulated cry
MODGR	Modulated groan
MODW	Modulated whistle
NGR(S)	Short N-shaped groan
P	Flat purr
P(A)	Ascending purr
P(MOD)(L)	Long modulated purr
P-AGR	Flat purr – Ascending groan
P-LWP	Flat purr – low whoop
PE-AM	Pulsed element- ascending moan
QK	Quack
SC	Flat scream
SC(L)	Long flat scream
SC-E	Screamy e
SQ	Flat squeak
TI(A)	Ascending trill
TI(MOD)(L)	Long, modulated trill
UC(S)-C(H)	Short, U-shaped cry – high cry
UC(S)-C(H)(S)	Short, U-shaped cry – high, short cry
WP	Whoop

3. Supplementary Figures

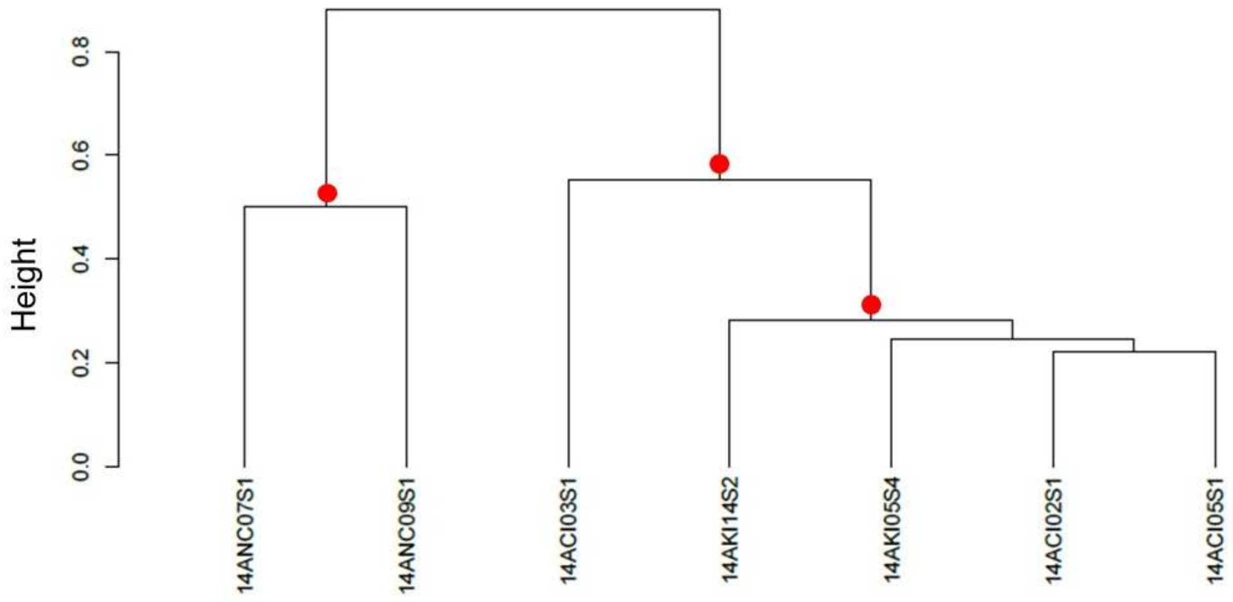

Figure S1. Dendrogram representing the similarity of the median sequence of units from theme 14 (song type 1b) for each individual singer recorded at the Kermadec Islands (KI) and the two wintering grounds in which these themes were present (New Caledonia: NC, and the Cook Islands: CI). The median string LSI scores were hierarchically clustered using average-linkage clustering and bootstrapped (n = 1000). The AU values (significant *p*-values > 95%, red dot [24, 32]) indicated the stability of each split in the tree. This was additionally confirmed using the Cophenetic Correlation Coefficient, which indicated that the structure of the tree was a very good representation of the associations present within the data (CCC= 0.91).

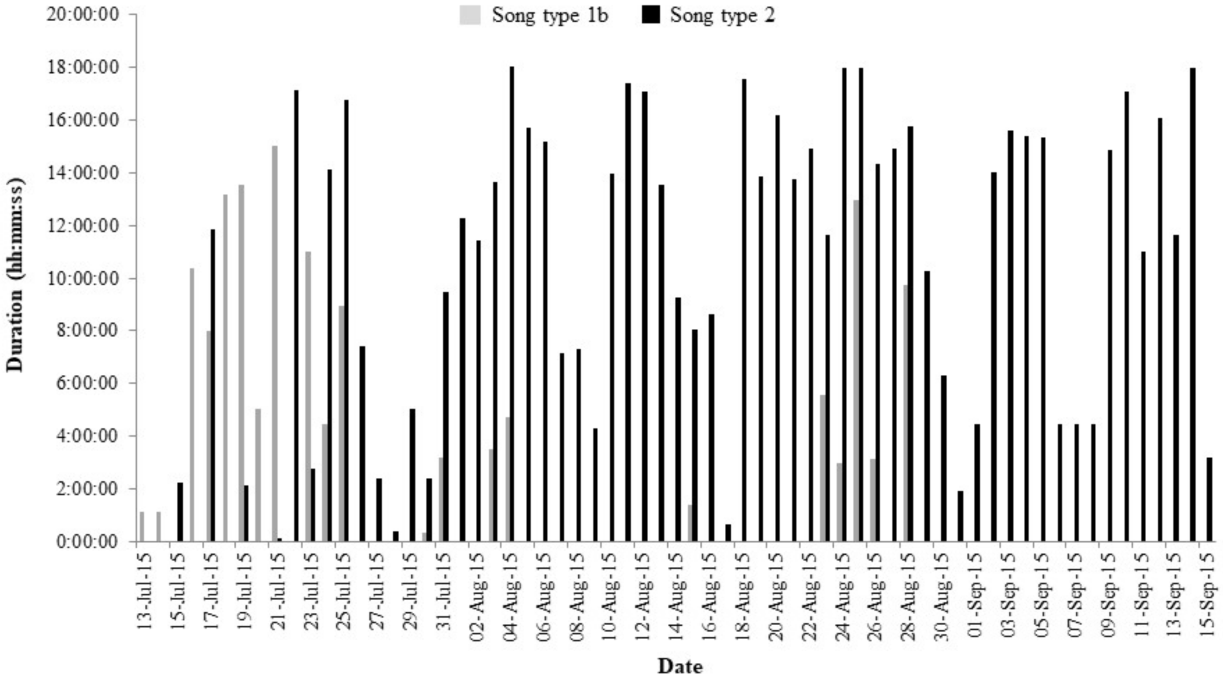

Figure S2. Hours per day song type 1b and song type 2 were recorded in New Caledonia between 13 July and 15 September 2015 by the SM2M+ Wildlife Acoustics recorder on an 18-hour duty (recording) cycle.

4. Supplementary Audio Files

Audio S1. Corresponding audio for song type 1a presented in figure 1.
Audio S2. Corresponding audio for song type 1b presented in figure 1.
Audio S3. Corresponding audio for song type 2 presented in figure 1.
Audio S4. Corresponding audio for song type 3 presented in figure 1.
